# Successful treatment of activated occult hepatitis B in a non-responder chronic hepatitis C patient

**DOI:** 10.1186/1743-422X-8-518

**Published:** 2011-11-14

**Authors:** Mohamed H Emara, Mohamed I Radwan

**Affiliations:** 1 Tropical Medicine Department, Faculty of Medicine, Zagazig University, Egypt

**Keywords:** occult hepatitis B, hepatitis C, pegylated interferon, lamivudine

## Abstract

We reported a 23 years old male with chronic hepatitis C virus infection, discontinued from pegylated interferon/ribavirin combination therapy due to a lack of early virological response. He has developed activation of occult hepatitis B virus that was successfully treated by a one year of lamivudine therapy.

## Background

Occult hepatitis B virus infection (OBI) is defined as the presence of hepatitis B virus (HBV) DNA, in serum and/or the liver tissue without detectable HBsAg with or without anti-HBc or anti-HBs outside the pre-seroconversion window period [1]. In chronic hepatitis C virus (HCV) infection, the presence of OBI has been associated with liver enzymes flare [2], increased severity of liver disease towards advanced fibrosis and cirrhosis [3], poor response to standard interferon-alpha in many [3,4], but not all [5] studies, and increased risk of hepatocellular carcinoma [6]. Concerning response to interferon therapy in chronic HCV a previous study by the first author found OBI to be a statistically non significant cause of interferon non-response [7].

## Case presentation

A 23 years old male patient with chronic HCV (discovered on a pre-employment checkup 2 years ago) was a candidate for the combination therapy (pegylated interferon/ribavirin) according to the guidelines of the National Committee for Control and Prevention of viral Hepatitis "C" in Egypt. He was not operated upon apart from circumcision; also he did not receive blood or blood product transfusion. He had no chronic medical diseases. His BMI was 27.5 and a liver biopsy score of A2 F2 (Metavir) without steatosis. His laboratory parameters are shown in table 1. Treatment schedule included pegylated interferon α 2a 180 ug/week (Pegferon, Roche, Switzerland) and ribavirin 1000 mg/day (Virin, Segma, Egypt). At week 12 of therapy he had his HCV RNA higher than the base line value (lack of early virologic response) and hence he was discontinued from the combination therapy. At that time his laboratory parameters showed OBI with HBV DNA values > 2000 IU (real time PCR technique with a detection limit of 12 IU, Roche Diagnostics, Switzerland) and a decision to treat this activation with lamivudine (available, safe, affordable) was taken. After a period of 6 months the patient was exposed to both HCV and HBV viral load assessment, HBV was undetected, while HCV RNA was decreased in comparison with the previous values. A decision to continue on lamivudine was taken. Six months later HBV DNA was assayed and still undetected. Since that time he discontinued lamivudine due to financial constraints. Six months after this discontinuation he was exposed to HBV and HCV viral load determination. While HBV was undetected, HCV RNA level was decreased to 7062 IU/ml.

**Table 1:** Laboratory characteristics of the patient

	Base line	Week 12 after interferon	Six months after Lamivudine therapy	Twelve months after lamivudine therapy	Six months after lamivudine discontinuation
ALT (IU/ml)	19 (12)	54 (30-65)	39 (30-65)	58 (40)	38 (40)
AST (IU/ml)	38 (12)	24 (15-37)	26 (15-37)	46 (40)	27 (40)
HCV RNA (IU/ml)	4174	14839	8970	Not done	7062
HBsAg	Negative	Negative	Not done	Not done	Negative
HBeAg	Not done	Negative	Not done	Not done	Negative
HBcAb					
Total	Not done	Negative	Not done	Not done	Negative
IgM	Not done	Negative	Not done	Not done	Negative
HBs Ab	Not done	Negative	Not done	Not done	Negative
HBV DNA (IU/ml)	Not done	2980	Undetected	Undetected	Undetected

## Discussion

It is not recommended to screen for OBI in chronic HCV before initiation of antiviral therapy [7] and that is why we do not routinely perform HBV DNA examination or serological markers assay [8] before initiation of antiviral therapy for chronic HCV. So we had only a baseline HBsAg for this patient. On follow up all serological markers remained negative (HBsAg, HBeAg, anti-HBc, Anti-HBs), this may point for a primary occult infection in this patient rather than a false occult infection. In the former a low dose of HBV infection may occur and establish the lymphatic system and later may invade the liver [9], while in the later defective laboratory techniques may not detect an antigenically modified HBsAg [10].

There is a reciprocal inhibition of replication between both HBV and HCV, with dominance of HCV inhibitory effect on HBV replication by its core protein [11], therefore HBV may flare up when the HCV virus is treated [12], and this may explain the slightly high levels of HBV DNA (> 2000 IU/ml) in this patient. But what is not completely understood is that this patient is a non-responder to interferon therapy, however HBV flared and HCV RNA increased than the base line values. And also the decrease in HCV RNA levels noticed when HBV DNA was undetected. This may not favor the reciprocal inhibition of replication between HBV and HCV in this case.

Both younger age and lack of biochemical flare (no increase in serum transaminases) could point to an immune tolerance state of HBV infection in this patient.

There is no consensus about management of OBI when HBV DNA levels are low [13], while when HBV DNA level exceeds 2000 IU [12], treatment may be introduced. Relying on liver biopsy, evidence of fibrosis was detected (F2), although it is due to HCV rather than HBV infection, a decision to treat this patient with HBV viral load > 2000 IU was taken as the presence of this OBI may favor progression of this fibrosis towards cirrhosis. Our patient continued on lamiviudine therapy for 1 year during this year he had his HBV DNA examination twice and both were undetected. Due to financial constraints this patient discontinued lamivudine, 6 months after this we examined for HBV and HCV viral load, HBV DNA was still undetected and HCV RNA level was 7062 IU/ml.

In HBeAg negative chronic HBV infection treatment should be continued until HBsAg clearance. While in HBeAg positive cases a 6 months treatment period after HBeAg seroconversion is recommended before discontinuation of therapy [12]. Our patient is negative for all HBV serological markers and hence no finite duration for therapy could be planned. HBV DNA clearance thus may be the therapeutic target in this patient. In our case a one year treatment with lamivudine seems to be successful to treat this seronegative OBI activation, with HBV DNA clearance is the primary end point.

## Conclusions

Check the presence of OBI when HCV therapy fails may be justifiable. Lamivudine could successfully be used in the treatment of occult hepatitis B activation.

## Consent

Written informed consent was obtained from the patient for publication of this Case Report and any accompanying data. A copy of the written consent is available for review by the Editor-in-Chief of this journal.

## List of abbreviations

OBI: Occult hepatitis B virus infection; HBV: hepatitis B virus; HCV: hepatitis C virus

## Competing interests

The authors declare that they have no competing interests.

## Authors' contributions

ME: carried out collection of patients' data, patient monitoring through the whole period of follow up and writing of the manuscript. MR: carried out reviewing patients' data, guiding follow up period, preparation of manuscript. Both authors read and approved the manuscript.
